# Considerations for Maintaining Family Diversity in Commercially Mass-Spawned Penaeid Shrimp: A Case Study on *Penaeus monodon*

**DOI:** 10.3389/fgene.2019.01127

**Published:** 2019-11-12

**Authors:** Andrew Foote, David Simma, Mehar Khatkar, Herman Raadsma, Jarrod Guppy, Greg Coman, Erika Giardina, Dean Jerry, Kyall Zenger, Nick Wade

**Affiliations:** ^1^ARC Research Hub for Advanced Prawn Breeding, James Cook University, Townsville, QLD, Australia; ^2^Centre for Sustainable Tropical Fisheries and Aquaculture, College of Science and Engineering, James Cook University, Townsville, QLD, Australia; ^3^Aquaculture Program, CSIRO Agriculture and Food, Queensland Bioscience Precinct, St. Lucia, QLD, Australia; ^4^Seafarms Group Ltd., Flying Fish Point, QLD, Australia; ^5^Sydney School of Veterinary Science, Faculty of Sciences, University of Sydney, Camden, NSW, Australia

**Keywords:** selective breeding, family skewing, genetic diversity, parentage assignment, single nucleotide polymorphisms, mass spawners, penaeid shrimp

## Abstract

Skewed family distributions are common in aquaculture species that are highly fecund, communally (mass) spawned, and/or communally reared. The magnitude of skews pose challenges for maintaining family-specific genetic diversity, as increased resources are required to detect individuals from underrepresented families, or reliably determine relative survival as a measure of family performance. There is limited understanding of family skews or changes in family proportion of communally reared shrimp under commercial rearing conditions and particularly how this may affect genotyping strategies to recover family performance data in breeding programs. In this study, three separate batches of shrimp*, Penaeus monodon*, were communally spawned and reared, and then sampled as larvae when ponds were stocked at 30 days of culture (DOC) and as juveniles from commercial ponds during harvest at 150 DOC. A total of 199 broodstock contributed to the 5,734 progeny that were genotyped with a custom multiplex single nucleotide polymorphism (SNP) panel, and family assignments were cross-referenced using two parentage assignment methods, CERVUS and COLONY. A total of 121 families were detected, with some families contributing up to 11% of progeny at 30 DOC and up to 18% of progeny at harvest. Significant changes were detected for 20% of families from 30 to 150 DOC, with up to a 9% change in relative contribution. Family skew data was applied in several models to determine the optimal sample size to detect families, along with the ability to detect changes in relative family contribution over time. Results showed that an order of magnitude increase in sampling was required to capture the lowest represented 25% of families, as well as significantly improve the accuracy to determine changes in family proportion from 30 to 150 DOC. Practical measures may be implemented at the hatchery to reduce family skews; a cost-effective measure may be to address the initial magnitude differences in viable progeny produced among families, by pooling equal quantities of hatched larvae from each family. This study demonstrates the relationships between skews in families under commercial conditions, the ability to accurately detect families, and the balance of sampling effort and genotyping cost in highly fecund species such as shrimp.

## Introduction

Effective breeding programs seek to maintain genetic diversity to avoid issues from inbreeding and allow long-term selection of desirable production traits. In contrast to terrestrial agriculture species, many aquaculture species are highly fecund, mass spawned, and communally reared, which results in skewed family contributions that pose challenges to breeding programs (Jerry et al., 2001; Jerry et al., 2006; Lind et al., 2010; Domingos et al., 2014; Harris et al., 2016; Zhu et al., 2017). Traditional breeding programs that maintain genetic diversity through independent family lines need to consider the magnitude of family skews and the impact on sampling effort to detect, evaluate, and select underrepresented families. In more advanced programs that use within-family and DNA-based genomic diversity, an understanding of family skewing will assist in establishing broad founder stocks, avoiding genetic bottlenecks, and influencing overall genetic diversity and sampling for rare traits in underrepresented families.

In most aquaculture species, it is impractical to track families identified from independent spawnings through physical tagging methods, due to their small body size and high fecundity. While it is possible to spawn and rear families individually at the hatchery, this requires significant resources and introduces additional environmentally induced rearing effects among families (Jerry et al., 2001; Vandeputte and Haffray, 2014). An effective alternative to trace and identify families is the use of genetic markers, including microsatellites and single nucleotide polymorphism (SNP) panels for parentage assignment. These genetic approaches have proven reliable to recover important genealogical information from communally reared families. Genetic markers have been used for parental assignment and family distributions in a number of species that have been communally reared such as the pearl oyster, *Pinctada maxima* (Lind et al., 2010), barramundi, *Lates calcarifer* (Frost et al., 2006; Domingos et al., 2014), Kuruma shrimp, *Marsupenaeus japonicus* (Jerry et al., 2006), white leg shrimp, *Litopenaeus vannamei* (Harris et al., 2016), and black tiger shrimp, *Penaeus monodon* (Henshall et al., 2014; Sellars et al., 2014; Zhu et al., 2017). Mass spawning approaches can select for a range of progeny grow-out traits as with individual-based approaches; however, the `cost-effectiveness of genotyping needs to be considered in mass spawning approaches. There are some limitations to mass spawning approaches where communal rearing may inhibit assessment of a family-level trait, such as horizontal disease transmission (Noble et al., 2019). In addition, reproductive traits such as fecundity could not be obtained rapidly in communal spawning approaches.

A number of selection strategies have been proposed in an effort to balance genotyping costs with selection intensity and retention of overall genetic diversity. In walk-back selection, individuals are first selected based on trait performance, and then markers are used to make the pedigree. Further selection can then be performed to maximize selection intensity while minimizing the number of selected individuals per family (Doyle and Herbinger, 1994; Vandeputte and Haffray, 2014). Both theoretical and empirical studies of highly fecund aquaculture species have previously demonstrated that walk-back selection provides a good trade-off between genotyping costs, selection intensity, and maintaining genetic diversity (Sonesson et al., 2010; Domingos et al., 2014). However, the utility of walk-back selection is still restricted to simple traits that can be measured in the selection candidates themselves.

A more recent approach using SNP genetic marker technology, termed genomic best linear unbiased selection (GBLUP), uses genomic estimated breeding values (GEBV) from a genomic relationship matrix (GRM), rather than a pedigree-based relationship matrix. Creating a relationship matrix based on genetic markers allows the true genomic relationship of relatives to be determined more accurately. For example, the relationship of two full-sibs may be between 0.4 and 0.6, rather than 0.5 from pedigree (Sonesson et al., 2010). As recently developed GBLUP methodologies utilize more accurate relationship data, the overall GEBV will have higher accuracy compared to conventional BLUP selection schemes, depending on heritability of the trait. However, genotyping and evaluation of large numbers of individuals is needed to capture both the within- and between-family genetic variances for the traits of interest. Thus, one of the main limitations implementing this method in aquaculture production species is the lack of knowledge around efficient sampling and genotyping of commercial populations with highly skewed family distributions.

The black tiger shrimp, *P. monodon*, is an example of a highly fecund aquaculture species, which commonly displays order of magnitude differences in the production of viable progeny from each spawn (Arnold et al., 2013). In the current study, three batches of *P. monodon* were communally spawned and reared in a commercial hatchery and grow-out ponds. The study determined family skews from three batches (separate spawning events) at two ages: 30 days of culture (DOC) prior to stocking into commercial grow-out ponds as well as 150 DOC at harvest age. Family relationships were determined using 120 SNP markers to evaluate significant changes in relative family survival, as a measure of survival performance. We also modeled the effects of different sample sizes on detecting significant changes from families. Further, we quantified the effect that empirically observed family skews have upon the sampling requirement when detecting candidates for selection. These scenarios were then overlaid on both traditional walk-back selection approach and GBLUP approaches.

## Marterials and Methods

### Animal Origin, Spawning, and Rearing

Wild *P. monodon* broodstock were sourced off the coast of Northern Territory, Australia, and transferred to a commercial hatchery at Flying Fish Point, QLD. Broodstock underwent routine commercial maturation: maintained in an indoor tank system at a density of 3 m^−2^, with flow through seawater maintained at 28±0.5°C and fed on a commercial maturation diet. A total of 678 potential broodstock parents were allowed to mate naturally within the tank, with any unmated females then artificially inseminated. Females were unilaterally eyestalk ablated and those with ripe ovaries (Tan-Fermin and Pudadera, 1989) placed into communal spawning tanks, with up to five other mated females at any given time. As spawners were pooled, there was no equalization of family contribution; there was also no equalization across spawning tanks. As broodstock were not tagged, the contributing parents were not known at the time of spawning; instead, genotyping would determine parents retrospectively. Spawned eggs underwent routine commercial washing prior to transfer into a hatching tank, and then hatched nauplii were harvested and transferred into larval rearing tanks (LRTs) where they would be reared on a commercial diet to 30 DOC. At 30 DOC, LRTs were pooled (Table 1), and progeny were stocked into commercial grow-out ponds, under routine commercial conditions at a density of approximately 45 m^−1^ until harvested at 150 DOC. The three separate batches of families were kept discrete, and the three batches of contributing parents, larval tanks, and grow-out ponds were tracked and sampled for genotyping and parental assignments (Table 1).

**Table 1:** Sample size, assignment rate, and structure of families at 30 days of culture (DOC), 150 DOC, and overall by batch.

Batch					30 DOC					150 DOC			
	Mothers	Fathers	Parents	Families	LRT	LRT proportion into pond (%)	Sample size	Assignment rate (%)	Families	Pond	Sample size	Assignment rate (%)	Families
1	47	39	86	54	a	56.6	564	99.5	52	A	470	99.8	40
					b	43.4	470	98.7	^				
					a	55.8	^	^	^	B	470	99.8	27
					b	44.2	^	^	^				
2	32	27	59	35	c	49.6	470	97	33	C	470	99.6	25
					d	50.4	470	98.9	^				
					c	52.8	^	^	^	D	470	100	27
					d	47.2	^	^	^				
3	27	27	54	33	e	19.6	235	99.1	31	E	470	98.5	31
					f	22	235	98.7	^				
					g	31.3	235	97.4	^				
					h	27.1	235	99.6	^				
					e	22.8	^	^	^	F	470	98.3	30
					f	25.8	^	^	^				
					g	28.1	^	^	^				
					h	23.3	^	^	^				

LRT, larval rearing tank. ^data as above.

### Genetic Sampling

The three batches of mass-spawned *P. monodon* were sampled at the two life stages (30 and 150 DOC), along with the broodstock, to determine family origin. All tissues for genetic analyses were fixed directly into RNAlater solution (Ambion) and stored at −20°C until the total nucleic acid (TNA) was extracted. The genetic material from each individual broodstock, as well as each individual progeny at both 30 and 150 DOC, was associated with a unique plate location and identification label, enabling tracking of individual tissue throughout the extraction, genotyping, and parental assignment process.

As individual broodstock contributing to the mass spawnings were not tagged and tracked, all potential male and female broodstock parents (678) from the contributing maturation tanks had pleopod tissue sampled for genotyping. However, the broodstock tissue was collected after spawning, and some contributing parents may not have been sampled if they died prior to sampling. Whole post-larvae were sampled from the LRTs at 30 DOC when they were transferred to the grow-out pond.

Each LRT from each batch was sampled immediately prior to pooling and stocking into the commercial grow-out ponds, with the 30 DOC family distributions calculated by factoring in the relative stocking proportions from each LRT to each grow-out pond. A total of 5,734 progeny were genotyped across the eight LRTs at 30 DOC and six ponds at 150 DOC (Table 1). Prior to harvesting the commercial pond at 150 DOC, individuals were randomly collected by cast net directly from the pond and immediately euthanized in an ice slurry, and the gill tissue sampled from each individual.

### Nucleic Acid Extraction and Genotyping

Tissue disruption was performed using a mechanical bead beater, with TNA (RNA and DNA) extracted from each sample individually using the magnetic bead based MagJet RNA Kit (Thermo Fisher Scientific) on a KingFisher Flex 96 automated DNA extractor (Thermo Fisher Scientific) according to the manufacturer’s protocols. TNA was collected by omitting the DNase digestion step of the extraction procedure. DNA concentration was normalized to 25 ng/µl and genotyped using an established panel of 120 SNP markers (Sellars et al., 2014) on the Sequenom mass-spectrometer platform (Australian Genome Research Facility).

### Family Assignment

Genotyping data was cleaned prior to family assignment by removing individuals and SNP with >95% fail rates. SNPs that failed across all the broodstock were removed from further analysis: 20 from batch 1 and 2 and 20 from batch 3. Family assignments were determined by cross-referencing the output from two software packages: CERVUS 3.0 (Kalinowski et al., 2007) and COLONY 2.0 (Jones et al., 2010). CERVUS assigned parentage was based on the pairwise likelihood comparison approach, and after likelihood scores were generated for the parents of each offspring, offspring were assigned to the parent with the highest logarithm of the odds (LOD) score. COLONY determined parentage and sib-ship was based on a multi-locus genotype and utilized a full-likelihood method. Three subsample groups, candidate father, candidate mother, and offspring samples, were assigned to their most likely family cluster, having compared the likelihood of numerous different cluster groupings. CERVUS assignment of offspring to their parent used significant LOD scores at a 97% confidence level. Genotyping error rate was the set to the program recommended 5%. Parentage assignments through COLONY were performed using a long, full-likelihood approach with very high precision. To test assignment success at assumed lower genotyping error rate, two separate colony analyses were performed using a 1% and 0.01% error rate.

Outputs from CERVUS and COLONY were aligned, and final assignment of an offspring occurred using the following rules: if CERVUS and COLONY assignment were matching and showed 97% confident trio delta value (≥1) and a high cluster probability (>0.7) respectively, the uniform result was accepted; when CERVUS did not match COLONY but CERVUS showed confident pair delta values (≥2) in both parental assignments, the CERVUS result was accepted as a final assignment; when CERVUS showed low confidence in the assignment of one candidate parent, but a confident trio delta value (≥1), the CERVUS assignment was used; if there was no confidence in the CERVUS assignment, high probability cluster assignment (>0.7) from COLONY were accepted as the final assignment; offspring for which both CERVUS and COLONY showed no confidence in their assignment were deemed as unassigned and excluded from further analysis.

### Relative Family Contribution

Using the final family assignment, the relative family contribution was initially calculated for each LRT and pond separately, by dividing the total number of individuals in a family by the total number of individuals sampled in the LRT or pond. For all the ponds, the relative family contribution at the point of stocking (p_30 DOC_) was calculated for each family using the following formula: $p_{30\;DOC}=\sum_{i=1}^{m}\;p\;\times \;P_{i}$ where *m* represented the number of LRT that contributed to the pond in question, *p* represented the proportion of the family within the LRT and P_i_ represented the proportion of progeny stocked from that LRT into the pond (Table 1). Individual family proportions at the point of stocking and the point of harvest were tested to be significantly different from zero using a two-tailed z-test. The standard error (*SE*) of proportion, $SE={\left(p\;\times \left(\frac{1-p}{n}\right)\right)}^{2}$ and the z value: $z=\;\frac{p}{SE}$ were computed, with *p* the proportion of the examined family and *n* the total number of samples in the pond at stocking or harvest. Families in which the relative contribution was found not to differ significantly from zero at both time points were excluded from further analyses, as the number of individuals in these families was not sufficient to assess statistical change.

The change in the proportion of a family from 30 DOC to 150 DOC was estimated using the difference of the two sample proportions as (p1-p2) and the standard error of the difference in the proportions as $SE=\sqrt{\frac{p1(1-p1)}{n1}+\frac{p2(1-p2)}{n2}}$ where *p1* and *n1* are the proportion of the family and the total number of animals sampled at 30 DOC, and *p2* and *n2* are the proportion of the family and the total number of animals sampled at 150 DOC. The 95 % confidence interval for the difference in the two proportions was computed as 
(p1−p2)±1.96*SE where1.96 is the value of standard normal variate for the 95% confidence interval.

To test the significance of the change in the proportion of the family from 30 DOC to 150 DOC, a z-score (z) was computed as z =(p1−p2)/SE , and the P-value for observing a sample statistic as *z* was obtained using a two tailed z-test.

### Sampling Effort Under Skewed Family Contributions

All of the analysis in this section was performed using the R software (R Core Team, 2014) and custom scripts. The extent of additional sampling needed under skewed conditions was quantified by comparing the sampling effort between simulated pond populations with equal family sizes and simulated pond populations representing the relative family contributions in 150 DOC pond samples. Families with a relative contribution not significantly different from zero at 150 DOC were excluded from the model pond populations; sample size estimates were interpolated from model graphs; empirically derived average body weights for each family at 150 DOC were simulated to display a normal distribution; and simulated individuals were assigned a unique identifier and family identifier. Genotyping effort was quantified and compared against three potential selection strategies: strategy 1 (S1), a walk-back selection approach, selecting the heaviest individuals until each family is detected at least once. Body weight was derived from the mean body weight and standard deviation derived from family weight data using the R software “Rnorm” function; strategy 4 (S4), a walk-back selection approach selecting until at least four of the heaviest individuals from each family were selected, and strategy 50 (S50), a family-based approach randomly sampling until at least 50 individuals from each family present were identified, to provide sample size estimates for future BLUP or GBLUP selection breeding schemes. Simulations were performed for each pond sample using the family skews identified for each. To estimate the effect of family skews on the sample sizes, S1, S4, and S50 were run with two family distributions, referred to as P_R_ and P_ALL_. The observed family skews were incorporated into the model (P_r_: **Supplementary Figure 1**; Table 1), while a scenario with even contribution was also modeled (P_ALL:_ **Supplementary Figure 1**). P_R_ and P_ALL_ of all six pond samples were simulated containing 2×10^5^, 3×10^5^, and 4×10^5^ individuals, to reflect different potential population sizes found in commercial ponds.

Each simulation (S1, S4, and S50) was replicated 10 times using all sizes of P_R_ and P_ALL_ of every pond present in the three batches. During S1 and S4, a sample of 10,000 potential selection candidates was taken from the respective simulated pond population to reflect practices described in the selection of other shrimp species (De Donato et al., 2005). Individuals in the sample were ranked based on their weight, and multiples of 50 subsamples were taken without replacement from the heaviest individuals until each family was identified at least once (S1) or until at least four individuals from each family were identified (S4). For S50, multiples of 100 randomly sampled individuals were sampled without replacement, directly from the simulated populations until at least 50 individuals from each family present in the population were identified. We display the sampling effort to capture the top 75% of represented families in addition to 100% of families in all three models, due to the substantial increased effort to capture the remaining 25% of represented families (Table 2).

**Table 2:** The sample size required to detect the top 75% and 100% of families for each model with skewed family contributions.

Model	Proportion of families selected (%)			
	75		100	
	Sample size		Sample size	
	Min	Max	Min	Max
S1	290	1,640	3,900	9,200
S4	600	2,750	4,700	9,700
S50	4,000	5,900	9,800	26,800

## Results

### Family Assignment

Overall, a total of 199 out of 678 potential parents contributed to the offspring sampled (Table 1). A total of 2,914 individuals were sampled at 30 DOC and 2,820 at 150 DOC, with an overall assignment rate of 98.6% at 30 DOC and 99.3% at 150 DOC (combining the CERVUS and COLONY outputs) (Table 2). The minimum assignment rate was 97% (LRT c), while the average assignment rate for each batch was 99.5%, 98.1%, and 98.6% in batches 1, 2, and 3 respectively (Table 1).

### Relative Family Contribution

**Figure 1:**
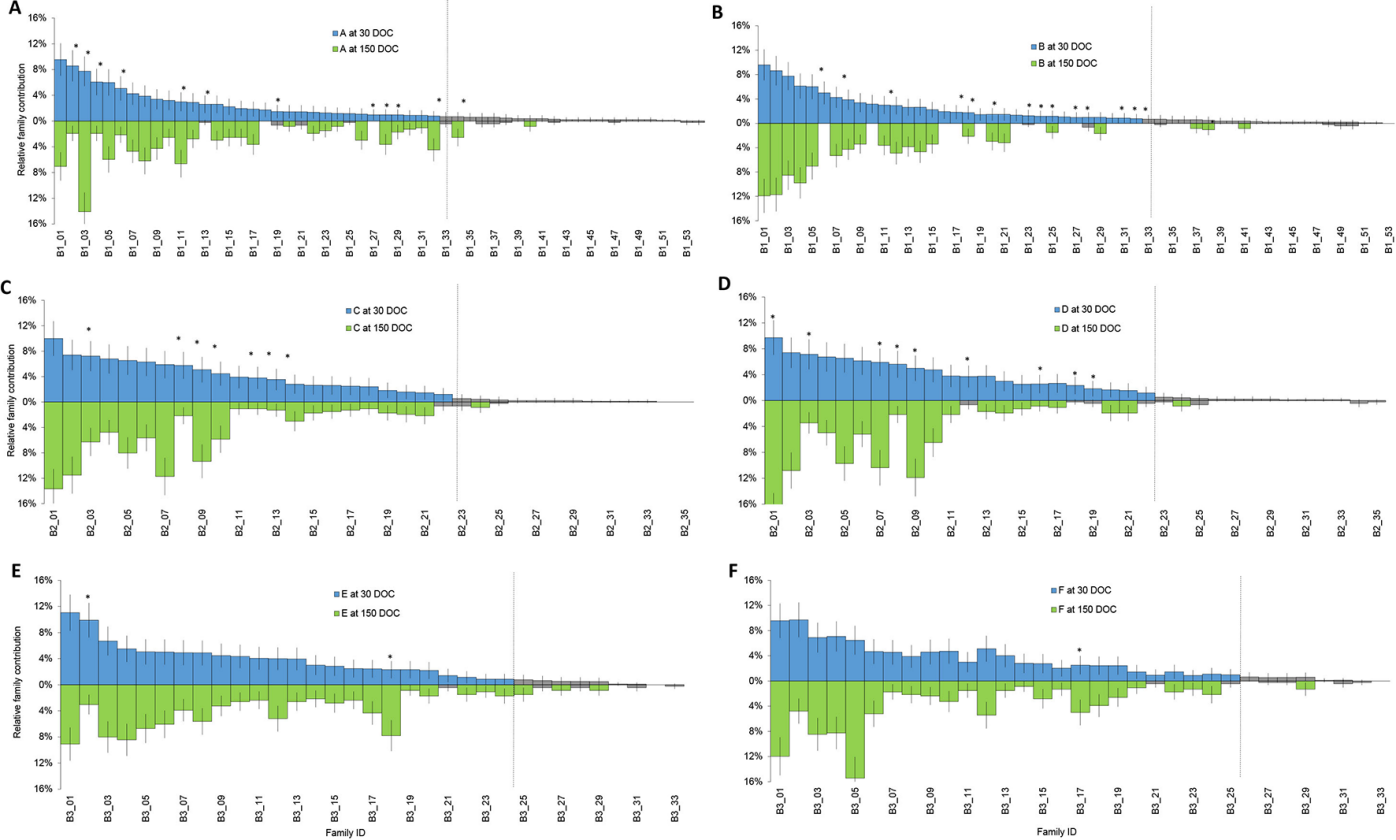
Relative proportion of each family at 30 DOC (stocking above the line in blue) and 150 DOC (corresponding families at harvest below the line in green) in ponds from batch 1 **(A** and **B)**; ponds from batch 2 **(C** and **D)** and ponds from batch 3 **(E** and **F)**. Bars show standard error. Grey bars represent families that were not significantly different from zero at 30 DOC or 150 DOC. Asterisks highlight families that showed a significant change in proportion from 30 DOC to 150 DOC.

Families that had a contribution significantly different from zero based on a two-tailed z-test were designated “top tier” for each batch (**Figure 1**, green), while the remainder of families had too few individuals to be reliably detected or further statistical analysis performed (**Figure 1**, grey). Overall, the number of top tier families were 33 of 54 families in batch 1, 22 of 35 families in batch 2, and 24 of 33 families in batch 3 (**Figure 1**).

In batch 1, relative contributions between 0 to 9.6% at 30 DOC (**Figure 1**, Blue) and between 0 to 14.1% at 150 DOC (**Figure 1**, Green). While All Top Tier Families Were Detected From Batch 1 (Ponds a and B), At 150 DOC, 4% of Families (N=2) Were Not Detected From Pond a and 22% From Pond B (N=12) At 150 DOC (**Figure 1**). in Batch 2, Relative Contributions Ranged Between 0% and 10% At 30 DOC and Between 0% and 17.7% At 150 DOC. All Top Tier Families Were Detected At Both 30 and 150 DOC (**Figures 1C, D**), and All Batch 2 Top Tier Families Were Detected Across Both Ponds B and C At 30 and 150 DOC (**Figure 1**). in Batch 3, Relative Contributions Ranged Between 0% and 11% At 30 DOC and Between 0% and 15.4% At 150 DOC (**Figures 1E, F**). Only 6% of Families (N = 2) Were Not Redetected At 150 DOC, and 3% of Families (N = 1) Were Only Detected At 150 DOC (With Two Individuals) (**Figure 1**).

### Changes in Relative Family Contribution

**Figure 2:**
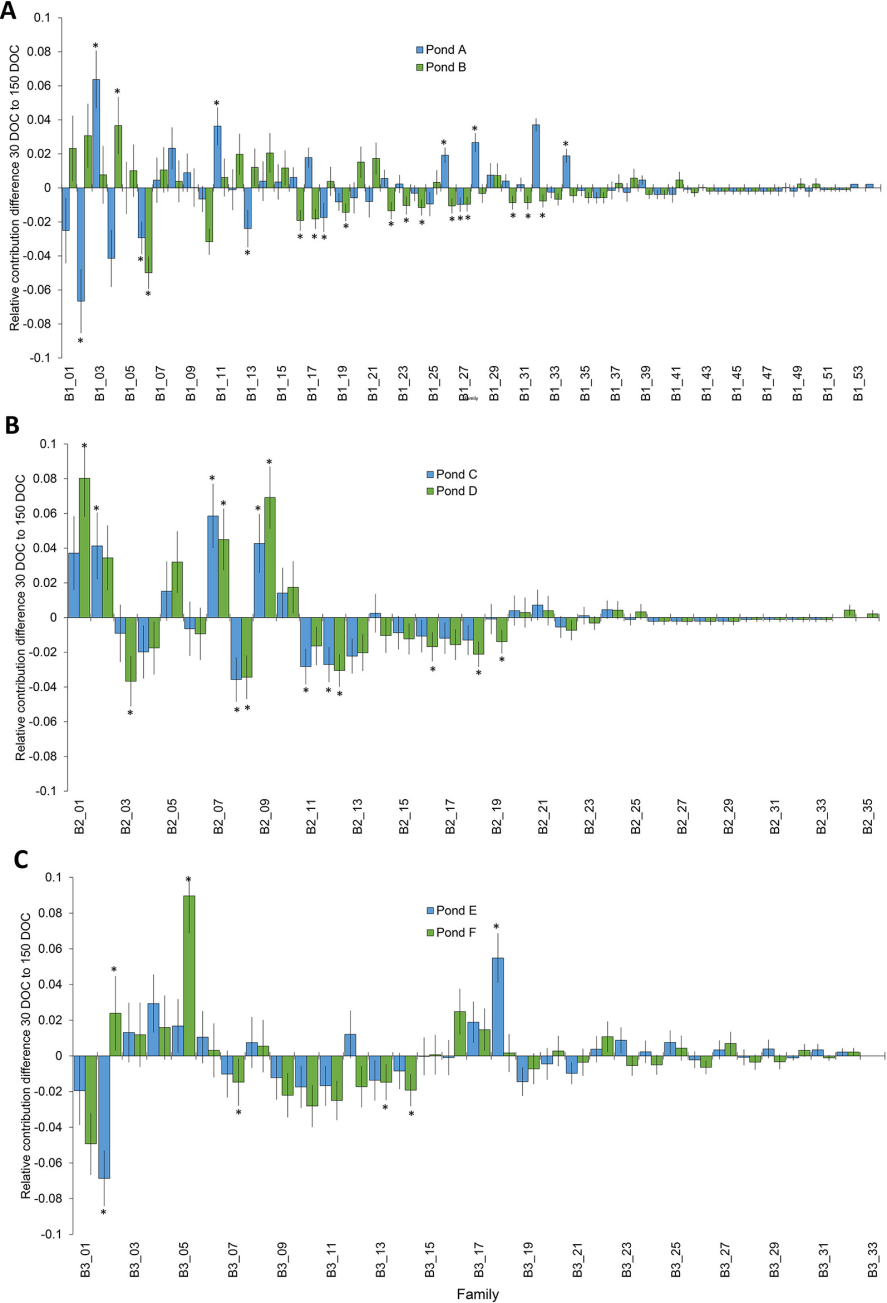
Change in the distribution of families from 30 DOC to 150 DOC from ponds A and B from batch 1 **(A)**; ponds B and C from batch 2 **(B)** and ponds D and E from batch 3 **(C)**. Asterisks highlight significant changes in proportion P<0.05.

**Figure 3:**
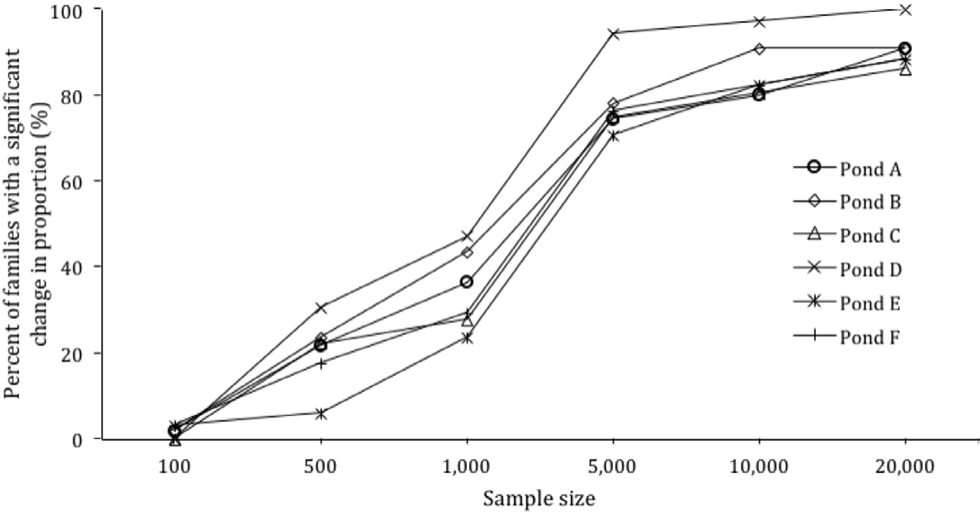
Power calculation for sample size versus percent of families with a significant change in proportion.

Significant changes in relative family contribution from 30 to 150 DOC were determined for each batch. In batch 1, changes to family contribution were detected in 22% of families (n=12) from pond A, and 26% of (n = 14) families from pond B (**Figure 2**), and ranged from a 6.7% decrease to a 6.4% increase (**Figure 2**). The majority of top tier families at 30 DOC remained in the top tier of represented families at 150 DOC: 93% in pond A and 87% in pond B (**Figure 2**). In batch 2, significant changes in relative family contribution were detected in 20% of families (n = 7) in pond C and 26% of families (n = 9) in pond D (**Figure 2**), and ranged from a 3.7% decrease to an 8% increase (**Figure 2**). Almost all 30 DOC top tier families remained in the top tier at 150 DOC, 95% (n = 21) in pond C and 91% (n = 20) in pond D. In batch 3, significant changes in relative family contribution were detected in only 6% of families (n = 2) in pond E and 15% of families (n = 5) in pond F (**Figure 2**), and ranged from a 6.9% decrease to a 9% increase (**Figure 2**). Almost all 30 DOC top tier families remained in the top tier at 150 DOC, 97% for ponds E and F. In all ponds across all batches, family rankings based on abundance were different at 150 DOC compared with 30 DOC (**Figure 3**), with relative abundance markedly different between ponds stocked from the same LRT.

### Modeling Genotyping Effort Under Skewed Family Contributions

Based on the skews identified in each pond, the effect of sample size on the ability to detect significant changes in family proportion was modeled. When the sample size per pond was increased from 500 to 1,000, 5,000, 10,000, and 20,000, the detection of significant changes in families from 30 to 150 DOC increased from 20.3% of families to 34.7%, 78.2%, 85.6%, and 90.7% of families respectively (**Figure 3**).

The relationship between the number of heaviest individuals selected and the proportion of families that reached the selection threshold of one (S1) and four (S4) followed a logarithmic trend in both types of pond populations (**Supplemetary Figure 1**). To capture every family present in the S1 or S4 models, up to 9,200 (4.60% of pond population) and 9,700 (4.85% of pond population) individuals were required, respectively (**Table 2**). For most ponds, the plateau at which increased sample size showed little benefit to detect additional changes in family contribution was estimated at 70% to 80% of individuals sampled. To capture significant changes in the remaining 20% to 30% of families was not benefited by increased sample sizes of 10,000 or 20,000 required increasing sampling effort, greatly decreasing the rate at which additional families passed the selection threshold (**Supplementary Figure 1** and **Table 2**). In the S1 and S4 models, sampling 290 (0.15% of pond population) and 600 (0.3% of pond population) of the heaviest individuals from ponds EALLS1 and EALLS4, respectively, captured the number of individuals required from 75% of families present (**Supplementary Figure 1** ****and **Table 2**). Ponds AS1, CS1, AS4, and CS4 required the highest amount of sampling to reach the 75% mark, at 1,640 (0.825% of pond population) and 2,750 (1.37% of pond population) of the heaviest individuals selected respectively. S50 populations were created *in silico* with equal and skewed family contributions; all populations displayed an exponential trend in the proportion of families with at least 50 individuals selected (**Supplementary Figure 1** and **Table 2**). Randomly sampling from populations with equal contributions required up to 2,600 individuals to reach 100% of families’ ponds (**Supplementary Figure 1**). To capture all the families in the S50 model, with the skewed family distributions observed, required up to 26,800 (13.4% of the pond population) to be sampled, while capturing the top 75% of families required a sample size of up to 5,900 individuals (2.95% of the pond population) (**Table 2**).

## Discussion

### Skewed Family Distributions

The sample size of approximately 500 individuals per pond, 1,000 per batch, at two commercially important time points for *P. monodon*, was adequate to detect and redetect all but the rarest of families. This enabled the skewness of family distributions to be determined, changes in relative contribution of families and differences in family rankings at harvest between ponds stocked with the same families. However, breeding programs focused on genetic improvement would typically benefit from increased sample sizes to improve the number of families detected and the accuracy of performance evaluations for each family. The benefits of increased sample sizes and sampling strategies as well as practical hatchery practices are discussed further below.

Sampling approximately 500 individuals per pond was sufficient to recapture all of the top tier represented families detected at pond stocking (30 DOC), again at harvest (150 DOC), in four of six ponds from two of three batches. When the two ponds from the remaining batch were combined, the batch sample size of approximately 1,000 was sufficient to capture all families in the top tier at both time points. The overall skewness of mass-spawned *P. monodon* in the current study increased from pond stocking (30 DOC), where relative family contributions were <1–11%, to harvest (150 DOC), where relative family contributions were <1–18%. The magnitude of family skews in the current study was similar to other aquaculture species including the penaeid shrimp *L. vannamei* (Harris et al., 2016) and *M. japonicus* (Jerry et al., 2006). The magnitude of change was slightly less than for other species such as the pearl oyster, *P. maxima*, after 150 DOC (Lind et al., 2010) and substantially smaller than the changes described for barramundi, *L. calcarifer* (Frost et al., 2006; Loughnan et al., 2013; Domingos et al., 2014), after 12 months of culture. Results demonstrated that differences in family skews, incorporating the effects specific to each species and the sampling time points in the production cycle, should be considered when determining sampling and genotyping effort to reliably determine family diversity within breeding programs.

To understand the relative impact of family skews and develop mitigation strategies, the underlying causes of skews needs to be determined. The skews at stocking for each batch (maximum 17.7% relative family contribution) was higher than the difference in skews from stocking to harvest for each batch (maximum 9% relative family contribution), indicating that initial factors such as variable broodstock fecundity and larval survival had more of an impact on family skews than differential survival during pond grow-out. Previous studies have reported that hatchery practices and differential family survival in that hatchery production stages can result in dramatic reductions in the effective population size (by greater than 70%) within a single generation (Frost et al., 2006; Harris et al., 2016). Practical strategies could be employed by hatcheries to lessen family skewing up to pond stocking, such as individually spawning and equal pooling of progeny per family at various time points in the hatchery. While pooling of families closer to the point of pond stocking will result in more equal family distributions, keeping families separate to trace family origin also has the potential to introduce a range of environmental rearing effects (Jerry et al., 2001). Physical tagging of individuals at the earliest life stages in the hatchery is impractical due to their small body size (Vandeputte and Haffray, 2014), and there are currently no other practical options to physically trace and equalize families without using separate rearing tanks (Jerry et al., 2001). Depending on the breeding program requirements, the best and most practical compromise might be to mitigate the variable fecundity, by spawning individually and equalizing contributions by pooling families once the progeny have hatched.

### Relative Survival

The change in relative family proportions from pond stocking to harvest can be used as a measure of relative family survival, allowing ranking of families and a potential selection criteria in breeding programs. While changes in relative family proportions from stocking to harvest were observed for many families, the sample size of approximately 500 per pond only resulted in detection of significant changes in proportion for 31% of the top tier and 20% of the total families. If this relative survival is to be used as a performance measure, the sample size would need to increase to allow detection of significant changes in a greater proportion of families. Increasing the sample size to 5,000, for example, would allow detection of significant changes in 78% of families. Cost-effective strategies to increase the quantity of individuals genotyped might be facilitated in the future through strategies such as DNA pooling that has been validated in other production animals such as chicken, beef cattle (Reverter et al., 2016), and sheep (Bell et al., 2017). DNA pooling allows significant cost savings with a number of pooled individuals genotyped together per sample (Henshall et al., 2012; Reverter et al., 2014).

There was little consistency between the relative survival of families, as well as the ranking of families by relative contribution across ponds, for each of the three batches. However, the majority of families in the top and bottom tiers of abundant families was consistent across ponds. This difference in family survival performance across ponds highlights the impact of environmental rearing effects, in addition to genetic effects. A future trial could stock families evenly across replicate ponds to discriminate the magnitude of genotype by environment (GxE) interactions influencing relative family survival (Dupont-Nivet et al., 2008) and their changes based on the genetic merit of the families.

A previous study on *M. japonicus* has shown GxE interactions directly affected growth rate in males and that selection of males based on performance in one environment might reduce the efficiency of potential breeding program (Jerry et al., 2006).

### Sampling Effort Under Skewed Family Contributions

Models for sampling effort demonstrated the impact of the family skews observed, with a doubling of sampling effort required to capture the lowest 25% of represented families, compared to capturing just the top 75% of families (**Table 2**). Capturing and monitoring genetic diversity and desirable traits in the top 75% of represented families might be suitable in certain breeding programs, providing a practical and efficient use of resources. However, consideration should also be given to the bottom 25% of families that might contain rare, desirable traits for selection. It is clear with the skews observed that sampling for the bottom 25% of families requires increased resources, alternative sampling strategies, and/or mitigation of skews at the hatchery.

The sampling effort, under the family skews observed, to detect the top 75% of families (minimum one individual per family: S1), through selection of the heaviest individuals, was compared to selecting four individuals per family (from the heaviest families: S4), to examine if sampling thresholds changed significantly. There was only a modest (66–100%) increase in genotyping effort required to increase the sampling threshold from one (S1) to four (S4) of the heaviest individuals per family (S1: 300–1,650 individuals and S4: 600–2,750 individuals). The selection threshold was described by a logarithmic curve, matching the trends reported for *M. japonicus* (Jerry et al., 2006) and *L. calcarifer* (Domingos et al., 2014). The proportional difference of heaviest individuals required between S1 and S4 was further reduced when sampling for 100% of the families: requiring an increase in sampling effort of just 5–21%. While S4 only required low to modest increases in sampling effort over S1, the model still assumes operation of a simplistic breeding scheme, only able to address traits that can be directly measured in selection candidates. For traits where this is not possible, more complex breeding schemes will require more than four candidates.

The S50 model was designed to provide sample size estimates for future BLUP or GBLUP selection breeding schemes. To assess the impact of skewed family contributions on sampling effort and to increase the accuracy of sample sizes estimates, the S50 model incorporated populations based on both equal family contribution and the relative family contributions derived from the six pond samples at 150 DOC. It has been previously demonstrated in *L. calcarifer* that skewed family contributions increased sampling effort significantly in comparison to cohorts with an even family contribution (Domingos et al., 2014). In the S50 model, equal family contribution had a similar effect: the curves describing the pond populations with equal contribution all followed an exponential trend of selecting 100% of families after ≤2,600 individuals were randomly sampled. In comparison, the skewed pond populations required 4,000–5,900 individuals to be randomly sampled to capture at least 50 individuals from 75% of families.

We evaluated the impact of the family skews on the sample size required under three different modes of selection. The models S1 and S4 used in this study were able to show that for *in silico* pond populations of *P. monodon*, the relationship between the number of the heaviest individuals sampled and the number of families fulfilling the selection criteria is described by a logarithmic trend, building upon previous published walk-back models in *L. calcarifer* (Domingos et al., 2014). Analysis of the models further revealed that the relationship for pond populations with equal family contributions was also described by a logarithmic trend, implying that another factor, such as family-specific differences in body weight distributions (Jerry et al., 2006), was influencing the number of heaviest individuals required to be sampled to capture family specific genetic diversity.

The novelty of the S50 model design was to provide a sample size estimate based on family skews identified in real pond samples for application in selection schemes, such as BLUP or GBLUP schemes that require at least 50 individuals from each family for accurate phenotype measurement within the training population. Results showed that selecting between 3,900 and 5,800 individuals was sufficient to capture 50 or more individuals from 75% of families across all ponds examined. In addition to the current study, the S50 model was designed to assist in the planning of BLUP and GBLUP selection schemes of other highly fecund and communally reared commercial aquaculture species.

### Family Assignment

This study used two complementary family assignment methods to confidently determine the family distribution of 5,670 *P. monodon* progeny on an established low-density 120 SNP marker panel, at a rate of 98.9%. The use of two software packages CERVUS and COLONY allowed cross-referencing of assignments, increased assignment rates, and decreased erroneous assignments, particularly when a parent was missing. CERVUS was able to provide confident assignment in families where both parents were identified as part of the sampled broodstock. However, in the current study, some broodstock were not sampled due to mortalities. In these cases, CERVUS identified the next most likely parent from the pool of broodstock sampled (Kalinowski et al., 2007) and often indicated incorrect assignment with low confidence. In these cases of missing parents, COLONY found no candidate parent that could fit the family cluster and thus marked the parent as unknown (Jones et al., 2010). COLONY allowed missing broodstock individuals to be identified on multiple occasions in all three batches, complementing the CERVUS results. When CERVUS showed no confidence in either parental assignment, COLONY allowed assignment of individuals to a respective family cluster with a high exclusion probability and overcame erroneous parental assignments.

While COLONY improved the rate and accuracy of assignments, its utility could have been improved further with a less stringent genotyping error rate, more suitable for the marker density of the SNP panel. The low genotyping error rates of 1% and 0.01% were too low for a marker density of 103–104 effective SNPs, allowing only for ≤1 genotyping errors across the entire SNP panel/individual. In some cases, this low margin of error resulted in COLONY erroneously assigning a parent as unknown. However, in these cases, the grouping of progeny was still consistent with the CERVUS output, and the effect on overall assignment success was negligible since CERVUS provided a confident parental assignment. Future studies should ensure the marker density of the SNP panel is considered when determining the genotyping error rate in software packages such as COLONY.

The overall assignment success in this study at 98.9% was comparable to a previous study on *P. monodon*, using the same 120 SNP markers at 99.8% (Sellars et al., 2014). However, the assignment rate of 98.9% in the current study included families with missing parents. When the previous *P. monodon* study included one missing parent, their correct assignment rate dropped to <15% and then <3% when both parents were missing (Sellars et al., 2014). The assignment rates that used 120 SNP markers, in this and other studies, were high relative to other studies on crustaceans that only used a small number of microsatellite markers. The previous *P. monodon* study that used the same SNP markers also made assignments using 13 microsatellite markers, with correct parentage assignments comparably low at 81.7% (Sellars et al., 2014), while another study with *P. monodon* achieved 89% using six microsatellite markers (Zhu et al., 2017). Assignment success on other penaeid shrimp using microsatellites markers have been reported at 82.3% in *L. vannamei* (Harris et al., 2016) and 91% in *M. japonicus* (Jerry et al., 2006).

The number of candidate broodstock can be reduced from a large pool of potentially contributing parents to a smaller pool or only the contributing parents, by tagging and tracking broodstock and their spawning contributions. In the current study, this could have reduced genotyping 678 potential candidate parents to 199 contributing parents. Furthermore, sampling candidate parents at or prior to mating for the males and at spawning for the females would ensure contributing broodstock are not missed. If all contributing parents are known, it would also significantly reduce the complexity of family assignments and the likelihood of incorrect assignments. Breeding programs for penaeids may either create mating pools where natural mating can only occur between parents in a given pool or specific mate pairings can be made directly (with or without half-sibs) through artificial insemination (AI). In both the tracked mating scenarios mentioned, accurate tracking of female molting must also be considered to eliminate contributions from unintended males prior to mate pools or following AI (as the female loses the spermatophore when molting). Reliable long-term tagging methods such as passive integrated transponder (PIT) tagging would allow sufficient time for selection criteria such as pedigree and viral loading to be determined and linked to the broodstock, allowing informed mating or culling decisions (Foote et al., 2018).

Inbreeding would also be more easily controlled in mass spawning approaches where broodstock are tagged and their pedigree/relatedness known, as this would allow informed mating crosses and tracking of contributions to progeny. However, it must be acknowledged that in a communal spawning and rearing approaches, it is more difficult to track smaller families, and they are more likely to be lost.

## Conclusions

Overall, skewed relative family contributions increased from pond stocking (30 DOC) to harvest age (150 DOC). However, skewness at pond stocking was greater than the increase in skewness during pond grow-out. Therefore, practical mitigation strategies implemented in the hatchery are likely to reduce overall skewing; however, the cost, practicality, and differential environmental rearing effects of the strategy implemented need to be considered. Equalizing and pooling family contributions after they have hatched as nauplii may be a cost-effective strategy. The change in relative family proportion from stocking to harvest age provided a survival measure and family ranking to be determined for each family, which could be incorporated as a selection criteria in breeding programs. Differences in survival and family rankings between ponds outline the importance of environmental effects on survival. The sample size of approximately 500 individuals genotyped per pond was sufficient, with the skewness observed, to detect most families at harvest age. However, larger sample sizes would allow greater numbers of individuals per family to be detected as well as increase the ability to detect families that showed a significant change in relative proportion. Models that incorporated the family skews demonstrated the magnitude increase in sampling required to detect the lowest 25% of families as well as their change in proportion during pond grow-out. Overall, this study provides the practical information on sampling effort to accurately and reliably detect families for future selective breeding programs in highly fecund aquacultured species including shrimp and recommendations on key actions to mitigate skews.

## Data Availability Statement

The raw data supporting the conclusions of this manuscript will be made available by the authors, without undue reservation, to any qualified researcher.

## Ethics Statement

This study was exempt from ethics committee approval as the invertebrate animals, shrimp, are not subject to ethics approval.

## Author Contributions

AF led the project and manuscript. AF, NW, GC, KZ, HR, and DJ were involved in concept design and delivery and manuscript revision. EG led animal rearing and harvesting. AF and NW led sampling and processing. AF, DS, and JG performed family assignments. MK and HR led statistical analysis and power calculations. DS, JG, and KZ led sample size modeling.

## Funding

This project was funded as part of the Australian Research Council Industrial Transformation Research Program, in collaboration with industry partners Seafarms Ltd. and the Australian Genome Research Facility.

## Conflict of Interest

Author EG was seconded from Seafarms Ltd., an industry participant in the ARC Hub for Advanced Prawn Breeding, and assisted with animal rearing and harvesting. Author EG and the company Seafarms Ltd. were not involved in the study design, analysis, interpretation of data, the writing of this article, or the decision to submit it for publication. 

The remaining authors declare that the research was conducted in the absence of any commercial or financial relationships that could be construed as a potential conflict of interest.

## Abbreviations

DOC, days of culture; LRT, larval rearing tank; SNP, single nucleotide polymorphism; LOD, logarithm of the odds; S1, strategy 1; S4, strategy 4; S50, strategy 50; GBLUP, genomic best linear unbiased selection; GEBV, genomic estimated breeding values; GRM, genomic relationship matrix.

## Supplementary Material

Supplementary Figure 1 The number of individuals required at harvest to be genotyped from a subsample (n= 10,000) of 12 populations (n=200,000), to include at least one offspring from each family, with individuals ranked and selected based upon highest weight. Black dashed line marks the minimum and red dashed line the maximum number of heaviest individuals required to capture at least one individual from 75% of families for model S1 **(A)**, S4 **(B)**, S50 **(C)**. ‘ALL’ indicates results from models using equal family contribution within the simulated pond population.

## References

[B1] Arnold S. J. Coman G. J. Emerenciano M. (2013). Constraints on seedstock production in eighth generation domesticated Penaeus monodon broodstock. Aquaculture 410–411, 95–100. 10.1016/j.aquaculture.2013.06.023

[B2] Bell A. M. Henshall J. M. Porto-Neto L. R. Dominik S. McCulloch R. Kijas J. (2017). Estimating the genetic merit of sires by using pooled DNA from progeny of undetermined pedigree. Genet. Sel. Evol. 49, 1–7. 10.1186/s12711-017-0303-8 28245804 PMC5331749

[B3] De Donato M. Manrique R. Ramirez R. Mayer L. Howell C. (2005). Mass selection and inbreeding effects on a cultivated strain of Penaeus (Litopenaeus) vannamei in Venezuela. Aquaculture 247, 159–167. 10.1016/j.aquaculture.2005.02.005

[B4] Domingos J. A. Smith-Keune C. Jerry D. R. (2014). Fate of genetic diversity within and between generations and implications for DNA parentage analysis in selective breeding of mass spawners: A case study of commercially farmed barramundi, Lates calcarifer. Aquaculture 424–425, 174–182. 10.1016/J.AQUACULTURE.2014.01.004

[B5] Doyle R. W. Herbinger C., (1994). “The use of DNA fingerprinting for high-intensity, within-family selection in fish breeding,” in Proceedings of the World Congress on Genetics Applied to Livestock Production, 364–371.

[B6] Dupont-Nivet M. Vandeputte M. Vergnet A. Merdy O. Haffray P. Chavanne H. (2008). Heritabilities and GxE interactions for growth in the European sea bass (Dicentrarchus labrax L.) using a marker-based pedigree. Aquaculture 275 (1–4), 81–87. 10.1016/j.aquaculture.2007.12.032

[B7] Foote A. R. Stratford C. N. Coman G. J. (2018). Passive integrated transponder (PIT) tagging black tiger shrimp, Penaeus monodon: Applications for breeding programs. Aquaculture 491, 321–324. 10.1016/j.aquaculture.2017.12.029

[B8] Frost L. A. Evans B. S. Jerry D. R. (2006). Loss of genetic diversity due to hatchery culture practices in barramundi (Lates calcarifer). Aquaculture 261, 1056–1064. 10.1016/J.AQUACULTURE.2006.09.004

[B9] Harris L. J. Sellars M. J. Perez F. (2016). Differential family-based survival of Pacific White shrimp during communal larval culture and implications for selective breeding programs. Aquac. Int. 24, 273–279. 10.1007/s10499-015-9924-5

[B10] Henshall J. M. Dierens L. Sellars M. J. (2014). Quantitative analysis of low-density SNP data for parentage assignment and estimation of family contributions to pooled samples. Genet. Sel. Evol. 46, 1–17. 10.1186/s12711-014-0051-y PMC4244062 25183297

[B11] Henshall J. M. Hawken R. J. Dominik S. Barendse W. (2012). Estimating the effect of SNP genotype on quantitative traits from pooled DNA samples. Genet. Sel. Evol. 44, 12. 10.1186/1297-9686-44-12 22507187 PMC3353226

[B12] Jerry D. R. Preston N. P. Crocos P. J. Keys S. Meadows J. R. S. Li Y. (2006). Application of DNA parentage analyses for determining relative growth rates of Penaeus japonicus families reared in commercial ponds. Aquaculture 254, 171–181. 10.1016/J.AQUACULTURE.2005.10.035

[B13] Jerry D. R. Purvis I. W. Piper L. R. (2001). Opportunities for genetic improvement in crustacean species. Proc. Assoc. Advmt. Anim. Breed. Genet. 14, 55–59.

[B14] Jones A. G. Small C. M. Paczolt K. A. Ratterman N. L. (2010). A practical guide to methods of parentage analysis. Mol. Ecol. Resour. 10, 6–30. 10.1111/j.1755-0998.2009.02778.x 21564987

[B15] Kalinowski S. T. Taper M. L. Marshall T. C. (2007). Revising how the computer program CERVUS accommodates genotyping error increases success in paternity assignment. Mol. Ecol. 16, 1099–1106. 10.1111/j.1365-294X.2007.03089.x 17305863

[B16] Lind C. E. Evans B. S. Taylor J. J. U. Jerry D. R. (2010). The consequences of differential family survival rates and equalizing maternal contributions on the effective population size (Ne) of cultured silver-lipped pearl oysters, Pinctada maxima. Aquac. Res. 41, 1229–1242. 10.1111/j.1365-2109.2009.02410.x

[B17] Loughnan S. R. Domingos J. A. Smith-Keune C. Forrester J. P. Jerry D. R. Beheregaray L. B. (2013). Broodstock contribution after mass spawning and size grading in barramundi (Lates calcarifer, Bloch). Aquaculture 404–405, 139–149. 10.1016/j.aquaculture.2013.04.014

[B18] Noble T. H. Coman G. J. Wade N. M. Thomson P. C. Raadsma H. W. Khatkar M. S. (2019). Genetic parameters for tolerance to gill-associated virus under challenge-test conditions in the black tiger shrimp (Penaeus monodon). Aquaculture 734428. 10.1016/j.aquaculture.2019.734428

[B19] R Core Team. (2014). R: A language and environment for statistical computing. Vienna, Austria: R Foundation for Statistical Computing. URL http://www.R-project.org/.

[B20] Reverter A. Henshall J. M. McCulloch R. Sasazaki S. Hawken R. Lehnert S. A. (2014). Numerical analysis of intensity signals resulting from genotyping pooled DNA samples in beef cattle and broiler chicken. J. Anim. Sci. 92, 1874–1885. 10.2527/jas.2013-7133 24663186

[B21] Reverter A. Porto-Neto L. R. Fortes M. R. S. McCulloch R. Lyons R. E. Moore S. (2016). Genomic analyses of tropical beef cattle fertility based on genotyping pools of Brahman cows with unknown pedigree. J. Anim. Sci. 94, 4096–4108. 10.2527/jas.2016-0675 27898866

[B22] Sellars M. J. Dierens L. Mcwilliam S. Little B. Murphy B. Coman G. J. (2014). Comparison of microsatellite and SNP DNA markers for pedigree assignment in Black Tiger shrimp, Penaeus monodon. Aquac. Res. 45, 417–426. 10.1111/j.1365-2109.2012.03243.x

[B23] Sonesson A. K. Meuwissen T. H. E. Goddard M. E. (2010). The use of communal rearing of families and DNA pooling in aquaculture genomic selection schemes. Genet. Sel. Evol. 42, 1–9. 10.1186/1297-9686-42-41 21092198 PMC3000837

[B24] Tan-Fermin J. D. Pudadera R. A. (1989). Ovarian maturation stages of the wild giant tiger prawn, Penaeus monodon Fabricius. Aquaculture 77(2–3), 229–242. 10.1016/0044-8486(89)90205-6

[B25] Vandeputte M. Haffray P. (2014). Parentage assignment with genomic markers: A major advance for understanding and exploiting genetic variation of quantitative traits in farmed aquatic animals. Front. Genet. 5, 1–8. 10.3389/fgene.2014.00432 25566319 PMC4264515

[B26] Zhu K. Yu W. Huang J. Zhou F. Guo H. Zhang N. (2017). Parentage determination in black tiger shrimp (Penaeus monodon) based on microsatellite DNA markers. Aquac. Int. 25, 827–836. 10.1007/s10499-016-0082-1

