# Supplementary figures and images for: Considerations for Maintaining Family Diversity in Commercially Mass-Spawned Penaeid Shrimp: A Case Study on Penaeus monodon
Source: Front Genet. 2019 Nov 12;10:1127. doi: 10.3389/fgene.2019.01127 (PMC6861421; doi:10.3389/fgene.2019.01127)

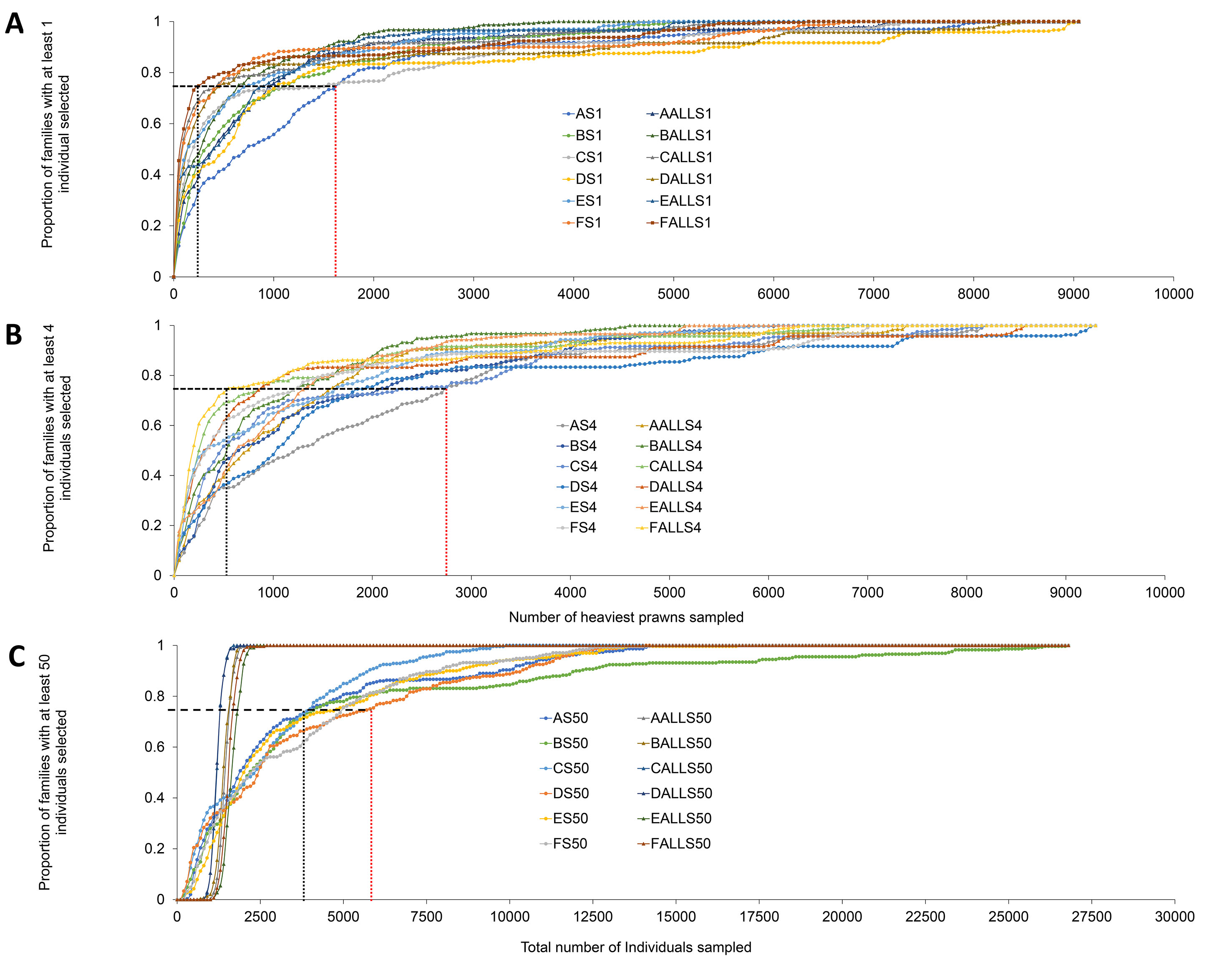

Supplement: Supplementary Figure 1 — The number of individuals required at harvest to be genotyped from a subsample (n= 10,000) of 12 populations (n=200,000), to include at least one offspring from each family, with individuals ranked and selected based upon highest weight. Black dashed line marks the minimum and red dashed line the maximum number of heaviest individuals required to capture at least one individual from 75% of families for model S1 (A), S4 (B), S50 (C). ‘ALL’ indicates results from models using equal family contribution within the simulated pond population. [file Image_1.jpeg]
